# Supplementary material for: Comparison of CRISPR-Cas9, CRISPR-Cas12f1, and CRISPR-Cas3 in eradicating resistance genes KPC-2 and IMP-4
Source: Microbiol Spectr. 2025 Apr 28;13(6):e02572-24. doi: 10.1128/spectrum.02572-24 (PMC12131857; doi:10.1128/spectrum.02572-24)
Supplement: Supplemental figures and tables — Figures S1 to S3 and Tables S1 to S8. [file spectrum.02572-24-s0001.docx]

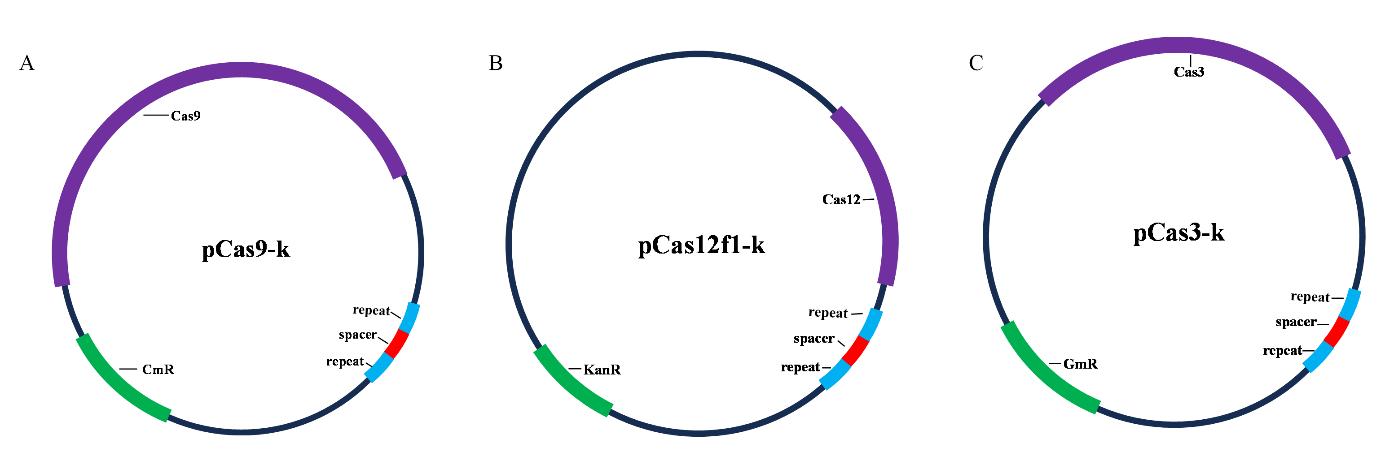


Figure S1 Plasmid map of three CRISPR systems targeted to KPC-2 and IMP-4 gene.

(A) The pCas9-k plasmid was constructed by inserting a spacer targeting KPC-2. Additional spacer fragments targeting either KPC-2 or IMP-4 were inserted to generate pCas9-k1, pCas9-k2, pCas9-i, pCas9-i1, and pCas9-i2. A similar approach was employed for the construction of pCas12f1-k (B) and pCas3-k (C). CmR denotes chloramphenicol resistance, KanR represents kanamycin resistance, and GmR indicates gentamicin resistance.


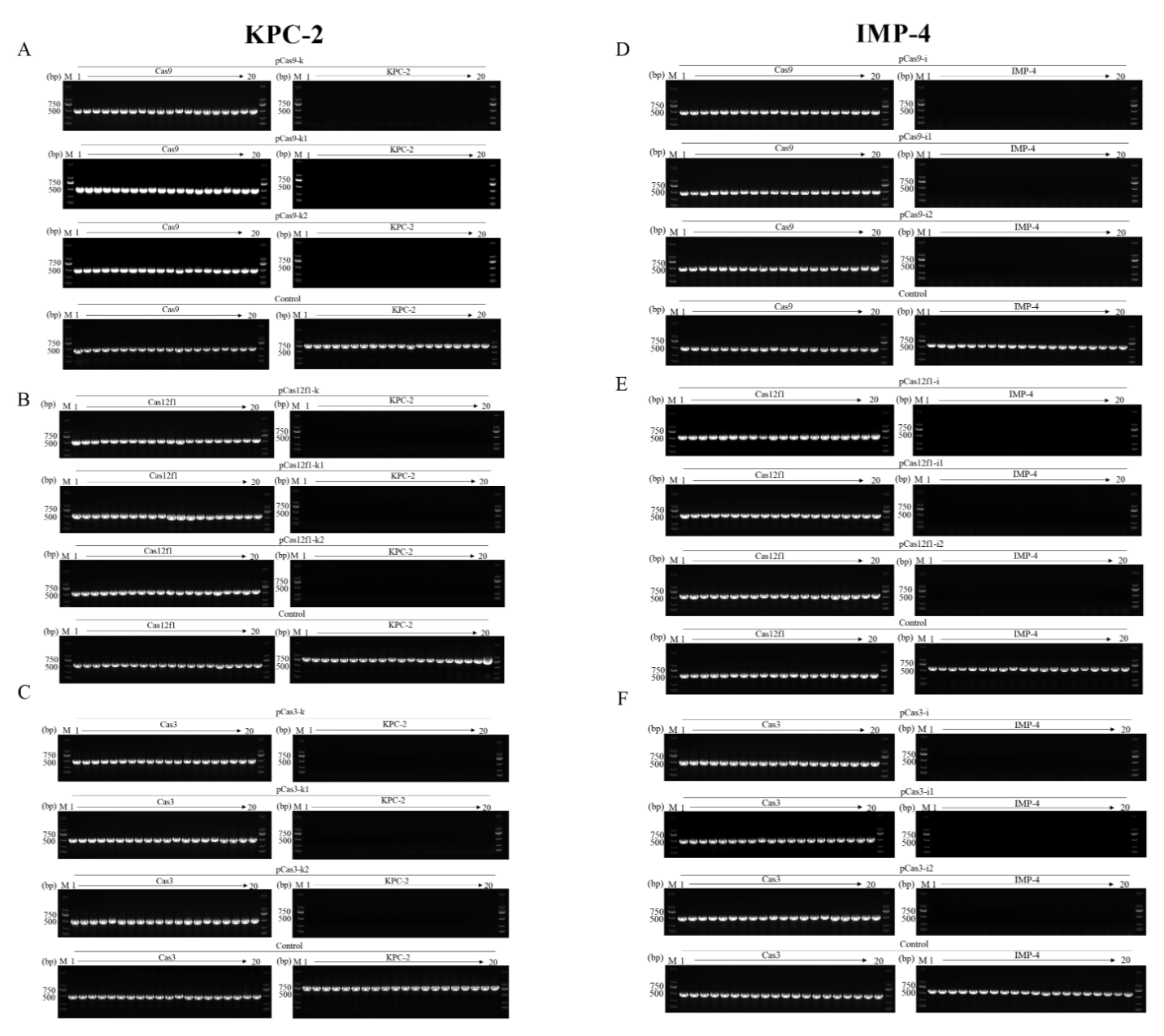


Figure S2 Confirmation of KPC-2/IMP-4 gene presence in *E. coli* DH5α+pKPC-2/pIMP-4 by PCR amplification for 20 single colonies. Control as a negative control is transformed into an empty vector plasmid. Lane M represents 2000 bp DNA molecular markers. KPC-2 was eliminated by CRISPR-Cas9 (A), CRISPR-Cas12f1 (B) and CRISPR-Cas3 (C). IMP-4 was eliminated by CRISPR-Cas9 (D), CRISPR-Cas12f1 (E) and CRISPR-Cas3 (F).


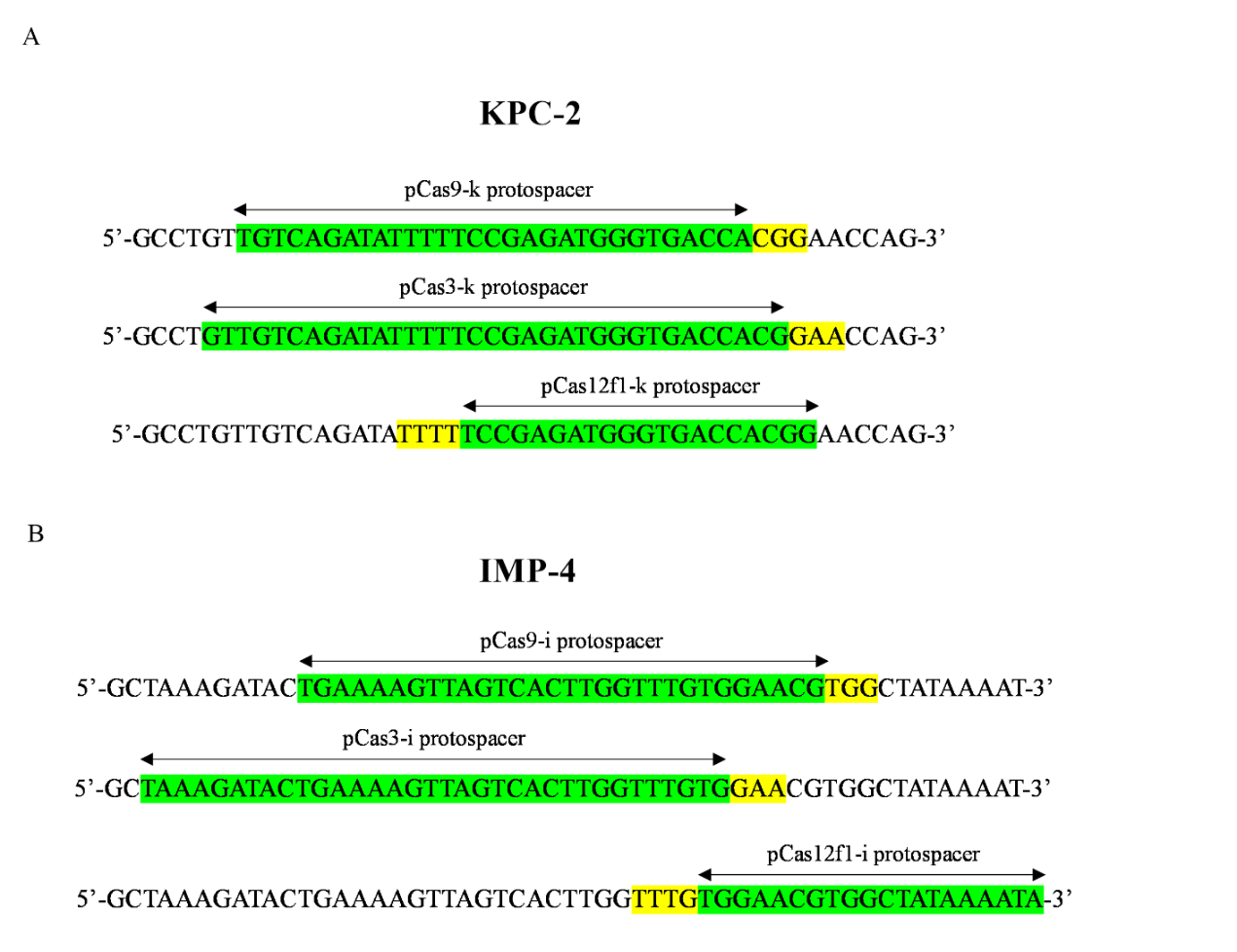


Figure S3 Three distinct CRISPR systems target the specific sites of the resistance genes KPC-2 and IMP-4, as well as their associated protospacer adjacent motifs (PAMs). (A) Three distinct CRISPR systems target the drug resistance gene KPC-2. (B) Three distinct CRISPR systems target the drug resistance gene IMP-4.

**Table S1** Bacterial strains and plasmids used in this study

| Bacterial strains or plasmids | Relevant characteristics | source |
| --- | --- | --- |
| *E. coli* DH5α | F-, φ80dlacZΔM15, Δ(lacZYA-argF) U169, deoR, recA1, endA1, hsdR17(rk-, mk+), phoA, supE44, λ-, thi-1, gyrA96, relA1 | TIANGEN, China |
| Plasmids |  |  |
| pSEVA551 | Tc^r^, expression vector | Laboratory stock |
| pSEVA551-KPC-2 | Tc^r^, recombinant vector derivative with KPC−2 gene | This study |
| pSEVA551-IMP-4 | Tc^r^, recombinant vector derivative with IMP-4 gene |  |
| pCas9 (Addgene,42876) | Cm^r^, tracRNA, gRNA, and cas9 expression plasmid | Laboratory stock (1-3) |
| pCas3cRh (Addgene,133773) | Gm^r^, tracRNA, gRNA, and cas3 expression plasmid |  |
| pCas12f1 | kan^r^, tracRNA, gRNA, and cas12f1 expression plasmid |  |
| pCas9-k | Cm^r^, pCas9 cloned with sgRNA targeting KPC−2 | This study |
| pCas9-k1 | Cm^r^, pCas9 cloned with sgRNA1 targeting KPC−2 |  |
| pCas9-k2 | Cm^r^, pCas9 cloned with sgRNA2 targeting KPC−2 |  |
| pCas3-k | Gm^r^, pCas3 cloned with sgRNA targeting KPC−2 |  |
| pCas3-k1 | Gm^r^, pCas3 cloned with sgRNA1 targeting KPC−2 |  |
| pCas3-k2 | Gm^r^, pCas3 cloned with sgRNA2 targeting KPC−2 |  |
| pCas12f1-k | kan^r^, pCas12f1 cloned with sgRNA targeting KPC−2 |  |
| pCas12f1-k1 | kan^r^, pCas12f1 cloned with sgRNA1 targeting KPC−2 |  |
| pCas12f1-k2 | kan^r^, pCas12f1 cloned with sgRNA2 targeting KPC−2 |  |
| pCas9-i | Cm^r^, pCas9 cloned with sgRNA targeting IMP-4 |  |
| pCas9-i1 | Cm^r^, pCas9 cloned with sgRNA1 targeting IMP-4 |  |
| pCas9-i2 | Cm^r^, pCas9 cloned with sgRNA2 targeting IMP-4 |  |
| pCas12f1-i | kan^r^, pCas12f1 cloned with sgRNA targeting IMP-4 |  |
| pCas12f1-i1 | kan^r^, pCas12f1 cloned with sgRNA1 targeting IMP-4 |  |
| pCas12f1-i2 | kan^r^, pCas12f1 cloned with sgRNA2 targeting IMP-4 |  |
| pCas3-i | Gm^r^, pCas3 cloned with sgRNA targeting IMP-4 |  |
| pCas3-i1 | Gm^r^, pCas3 cloned with sgRNA1 targeting IMP-4 |  |
| pCas3-i2 | Gm^r^, pCas3 cloned with sgRNA2 targeting IMP-4 |  |

**Note:** Tc^r^ denotes tetracycline resistance, Cm^r^ denotes chloramphenicol resistance, Kan^R^ represents kanamycin resistance, and Gm^R^ indicates gentamicin resistance.

**Table S2** Primers used in this study

| **Primer name** | **Primer sequence（5’-3’）** | **Purpose** |
| --- | --- | --- |
| KPC-2-F | CCTCGCTGTGCTTGTCATCC | PCR of KPC-2 deletion identification |
| KPC-2-R | GCCGTCTAGTTCTGCTGTCTT |  |
| IMP-4-F | AAAACTTGATGAAGGCGTTTATGTT | PCR of IMP-4 deletion identification |
| IMP-4-R | AATGTAAGTTTCAAGAGTGATGCG |  |
| pCas9-F | ACGCATTGATTTGAGTCAGCTA | PCR of pCas9 plasmid identification |
| pCas9-R | CATAGTGACTGGCGATGCTG |  |
| pCas3-F | GTCGATTTTTCAAGATACAGCGTG | PCR of pCas3 plasmid identification |
| pCas3-R | ATTTACCCAAGTCATGCAACAGAC |  |
| pCas12f1-F | CCTTTTTGCGTGTGATGCGA | PCR of pCas12f1 plasmid identification |
| pCas12f1-R | AACGCAGATTACAACGCAGC |  |
| KPC-2-qPCR-F | TCGTGTTTCCCTTTAGCC | qPCR of KPC-2 quantity of expression |
| KPC-2-qPCR-R | CCTTCATGCGCTCTATCG |  |
| KPC-2-qPCR-Probe | FAM-TTCAGCTCCAGCTCCCAGCG-BHQ1 |  |
| IMP-4-qPCR-F | GGATGGATTGAGAATTAAG | qPCR of IMP-4 quantity of expression |
| IMP-4-qPCR-R | GATGCTGAAGCTTATCTA |  |
| IMP-4-qPCR-Probe | FAM-ATAGCCACGTTCCACAAACC-BHQ1 |  |
| 16S-qPCR-F  16S-qPCR-R | CCAAGATCGAGGAGAATG  TTGCGAAGACTATTGATGA | qPCR of 16S quantity of expression |
| 16S-qPCR-Probe | FAM-CGCTTCTCCATCATCCACGG-BHQ1 |  |
| pCas9-k-F | AAACTGTCAGATATTTTTCCGAGATGGGTGACCAG | pCas9 spacer targeting KPC-2 |
| pCas9-k-R | AAAACTGGTCACCCATCTCGGAAAAATATCTGACA |  |
| pCas9-k1-F | AAACCTTTAGCCAATCAACAAACTGCTGCCGCTGG | pCas9 spacer1 targeting KPC-2 |
| pCas9-k1-R | AAAACCAGCGGCAGCAGTTTGTTGATTGGCTAAAG |  |
| pCas9-k2-F | AAACAATTGGCGGCGGCGTTATCACTGTATTGCAG | pCas9 spacer2 targeting KPC-2 |
| pCas9-k2-R | AAAACTGCAATACAGTGATAACGCCGCCGCCAATT |  |
| pCas3-k-F | GAAACCGTGGTCACCCATCTCGGAAAAATATCTGACAACG | pCas3 spacer targeting KPC-2 |
| pCas3-k-R | GAGACGTTGTCAGATATTTTTCCGAGATGGGTGACCACGG |  |
| pCas3-k1-F | GAAACCGTCTGGACCGCTGGGAGCTGGAGCTGAACTCCGG | pCas3 spacer1 targeting KPC-2 |
| pCas3-k1-R | GCGACCGGAGTTCAGCTCCAGCTCCCAGCGGTCCAGACGG |  |
| pCas3-k2-F | GAAACATGCGCTCTATCGGCGATACCACGTTCCGTCTGGG | pCas3 spacer2 targeting KPC-2 |
| pCas3-k2-R | GCGACCCAGACGGAACGTGGTATCGCCGATAGAGCGCATG |  |
| pCas12f1-k-F | GAACTCCGAGATGGGTGACCACGG | pCas12f1 spacer targeting KPC-2 |
| pCas12f1-k-R | GGCCCCGTGGTCACCCATCTCGGA |  |
| pCas12f1-k1-F | GAACCCGTGCCATACACTCCGCAG | pCas12f1 spacer1 targeting KPC-2 |
| pCas12f1-k1-R | GGCCCTGCGGAGTGTATGGCACGG |  |
| pCas12f1-k2-F | GAACTAAGCTTTCCGTCACGGCGC | pCas12f1 spacer2 targeting KPC-2 |
| pCas12f1-k2-R | GGCCGCGCCGTGACGGAAAGCTTA |  |
| pCas9-i-F | AAACTGAAAAGTTAGTCACTTGGTTTGTGGAACGG | pCas9 spacer targeting IMP-4 |
| pCas9-i-R | AAAACCGTTCCACAAACCAAGTGACTAACTTTTCA |  |
| pCas9-i1-F | AAACTGAATTAACTAATGAGCTGCTTAAAAAAGAG | pCas9 spacer1 targeting IMP-4 |
| pCas9-i1-R | AAAACTCTTTTTTAAGCAGCTCATTAGTTAATTCA |  |
| pCas9-i2-F | AAACAGTTAAAAATAAAATTGAAGTTTTTTATCCG | pCas9 spacer2 targeting IMP-4 |
| pCas9-i2-R | AAAACGGATAAAAAACTTCAATTTTATTTTTAACT |  |
| pCas3-i-F | GAAACCACAAACCAAGTGACTAACTTTTCAGTATCTTTAG | pCas3 spacer targeting IMP-4 |
| pCas3-i-R | GCGACTAAAGATACTGAAAAGTTAGTCACTTGGTTTGTGG |  |
| pCas3-i1-F | GAAACAGGCAGCCAAACTACTAGGTTATCTGGAGTGTGTG | pCas3 spacer1 targeting IMP-4 |
| pCas3-i1-R | GCGACACACACTCCAGATAACCTAGTAGTTTGGCTGCCTG |  |
| pCas3-i2-F | GAAACTAAATTTGCGTCACCCAAATTACCTAGACCGTACG | pCas3 spacer2 targeting IMP-4 |
| pCas3-i2-R | GCGACGTACGGTCTAGGTAATTTGGGTGACGCAAATTTAG |  |
| pCas12f1-i-F | GAACTGGAACGTGGCTATAAAATA | pCas12f1 spacer targeting IMP-4 |
| pCas12f1-i-R | GGCCTATTTTATAGCCACGTTCCA |  |
| pCas12f1-i1-F | GAACGCGGGGTTAACTATTGGCTA | pCas12f1 spacer1 targeting IMP-4 |
| pCas12f1-i1-R | GGCCTAGCCAATAGTTAACCCCGC |  |
| pCas12f1-i2-F | GAACGCTGCCTGAAAGGAAAATAT | pCas12f1 spacer2 targeting IMP-4 |
| pCas12f1-i2-R | GGCCATATTTTCCTTTCAGGCAGC |  |
| KPC-2(Kpn Ⅰ)-F | GG**GGTACC**TCGCTGTGCTTGTCATCCTT | PCR of KPC-2 fragment amplification |
| KPC-2(Sal Ⅰ)-R | GC**GTCGAC**TGTCTTGTCTCTCATGGCCG |  |
| IMP-4(Kpn Ⅰ)-F | GG**GGTACC**AAAACTTGATGAAGGCGTTTATGTT | PCR of IMP-4 fragment amplification |
| IMP-4(Sal Ⅰ)-R | GC**GTCGAC**AATGTAAGTTTCAAGAGTGATGCG |  |

**Note:** The underline indicates the protective bases, and the bold indicates the enzyme cutting site.

**Table S3** KPC-2-mediated drug resistance elimination by CRISPR-Cas9 system

| Antibiotics | Control | pCas9-k |
| --- | --- | --- |
| Amoxycillin-clavalanic acid  Ampicillin | >16/8, R*  >16, R | <=4/2, S*  <=4, S |
| Ampicillin-sulbactam | >16/8, R | <=4/2, S |
| Aztreonam | 16, R | <=4, S |
| Piperacillin | 64, R | <=4, S |
| Piperacillin-tazobactam | >32/4, R | <=4/4, S |

S*：sensitive R*：resistant

Control was used as negative control transferred into the empty plasmid

pCas9-k was used as experiment group transferred into the recombinant plasmid

**Table S4** KPC-2-mediated drug resistance elimination by CRISPR-Cas12f1 system

| Antibiotics | Control | pCas12f1-k |
| --- | --- | --- |
| Amoxycillin-clavalanic acid  Ampicillin | >16/8, R*  >16, R | <=4/2, S*  <=4, S |
| Ampicillin-sulbactam | >16/8, R | <=4/2, S |
| Aztreonam | 16, R | <=4, S |
| Piperacillin | 64, R | <=4, S |
| Piperacillin-tazobactam | >32/4, R | <=4/4, S |

S*：sensitive R*：resistant

Control was used as negative control transferred into the empty plasmid

pCas12f1-k was used as experiment group transferred into the recombinant plasmid

**Table S5** KPC-2-mediated drug resistance elimination by CRISPR-Cas3 system

| Antibiotics | Control | pCas3-k |
| --- | --- | --- |
| Amoxycillin-clavalanic acid  Ampicillin | >16/8, R*  >16, R | <=4/2, S*  <=4, S |
| Ampicillin-sulbactam | >16/8, R | <=4/2, S |
| Aztreonam | 16, R | <=4, S |
| Piperacillin | 64, R | <=4, S |
| Piperacillin-tazobactam | >32/4, R | <=4/4, S |

S*：sensitive R*：resistant

Control was used as negative control transferred into the empty plasmid

pCas3-k was used as experiment group transferred into the recombinant plasmid

**Table S6** IMP-4-mediated drug resistance elimination by CRISPR-Cas9 system

| Antibiotics | Control | pCas9-i |
| --- | --- | --- |
| Amoxycillin-clavalanic acid  Ampicillin | >16/8, R*  16, R | <=8/4, S*  <=4, S |
| Cefotaxime | 16, R | <=1, S |
| Ceftazidime | >16, R | <=1, S |

S*：sensitive R*：resistant

Control was used as negative control transferred into the empty plasmid

pCas9-i was used as experiment group transferred into the recombinant plasmid

**Table S7** IMP-4-mediated drug resistance elimination by CRISPR-Cas12f1 system

| Antibiotics | Control | pCas12f1-i |
| --- | --- | --- |
| Amoxycillin-clavalanic acid  Ampicillin | >16/8, R*  16, R | <=8/4, S*  <=4, S |
| Cefotaxime | 16, R | <=1, S |
| Ceftazidime | >16, R | <=1, S |

S*：sensitive R*：resistant

Control was used as negative control transferred into the empty plasmid

pCas12f1-i was used as experiment group transferred into the recombinant plasmid

**Table S8** IMP-4-mediated drug resistance elimination by CRISPR-Cas3 system

| Antibiotics | Control | pCas3-i |
| --- | --- | --- |
| Amoxycillin-clavalanic acid  Ampicillin | >16/8, R*  16, R | <=8/4, S*  <=4, S |
| Cefotaxime | 16, R | <=1, S |
| Ceftazidime | >16, R | <=1, S |

S*：sensitive R*：resistant

Control was used as negative control transferred into the empty plasmid

pCas3-i was used as experiment group transferred into the recombinant plasmid

**Reference**

1. Jiang W, Bikard D, Cox D, Zhang F, Marraffini LA. 2013. RNA-guided editing of bacterial genomes using CRISPR-Cas systems. Nature Biotechnology 31:233-239.

2. Csörgő B, León LM, Chau-Ly IJ, Vasquez-Rifo A, Berry JD, Mahendra C, Crawford ED, Lewis JD, Bondy-Denomy J. 2020. A compact Cascade-Cas3 system for targeted genome engineering. Nature Methods 17:1183-1190.

3. Wang Y, Sang S, Zhang X, Tao H, Guan Q, Liu C. 2021. Efficient Genome Editing by a Miniature CRISPR-AsCas12f1 Nuclease in Bacillus anthracis. Frontiers In Bioengineering and Biotechnology 9:825493.
